# FOXC2 expression and epithelial–mesenchymal phenotypes are associated with castration resistance, metastasis and survival in prostate cancer

**DOI:** 10.1002/cjp2.142

**Published:** 2019-10-01

**Authors:** Astrid Børretzen, Karsten Gravdal, Svein A Haukaas, Christian Beisland, Lars A Akslen, Ole J Halvorsen

**Affiliations:** ^1^ Centre for Cancer Biomarkers CCBIO, and Gade Laboratory for Pathology, Department of Clinical Medicine University of Bergen Bergen Norway; ^2^ Department of Pathology Haukeland University Hospital Bergen Norway; ^3^ Department of Clinical Medicine University of Bergen Bergen Norway; ^4^ Department of Urology Haukeland University Hospital Bergen Norway

**Keywords:** prostate cancer, FOXC2, EN‐switch, epithelial–mesenchymal transition, epithelial–mesenchymal plasticity, partial EMT, MET, castration resistance, survival

## Abstract

Epithelial–mesenchymal transition (EMT) is important for tumour cell invasion and metastasis and is a feature of aggressive carcinomas. EMT is characterised by reduced E‐cadherin and increased N‐cadherin expression (EN‐switch), and increased expression of the EMT‐regulating transcription factor Forkhead box protein C2 (FOXC2) has been associated with progression and poor prognosis in various malignancies. FOXC2 was recently highlighted as a novel therapy target in prostate cancer, but survival data on FOXC2 are lacking. This study evaluates the expression of FOXC2, E‐cadherin and N‐cadherin in different prostatic tissues focusing on EMT, clinico‐pathological phenotype, recurrence and patient survival. Tissue microarray sections from 338 radical prostatectomies (1986–2007) with long and complete follow‐up, 33 castration resistant prostate cancers, 33 non‐skeletal metastases, 13 skeletal metastases and 41 prostatic hyperplasias were stained immunohistochemically for FOXC2, E‐cadherin and N‐cadherin. FOXC2 was strongly expressed in primary carcinomas, including castration resistant tumours and metastatic lesions as compared to benign prostatic hyperplasia. A hybrid epithelial–mesenchymal phenotype, with co‐expression of E‐cadherin and N‐cadherin, was found in the majority of skeletal metastases and in a substantial proportion of castration resistant tumours. In localised carcinomas, the EN‐switch was associated with adverse clinico‐pathological variables, such as extra‐prostatic extension, high pathological stage and lymph node infiltration. In univariate survival analyses of the clinically important, large subgroup of 199 patients with Gleason score 7, high FOXC2 expression and EN‐switching were significantly associated with shorter time to clinical recurrence, skeletal metastases and cancer specific death. In multivariate Cox' survival analysis, high FOXC2 and the EN‐switch, together with Gleason grade group (GG3 versus GG2), were independent predictors of time to these end‐points. High *FOXC2* gene expression (mRNA) was also related to patient outcome, validating our immunohistochemical findings. FOXC2 and factors signifying EMT or its intermediate states may prove important as biomarkers for aggressive disease and are potential novel therapy targets in prostate cancer.

## Introduction

Prostate cancer is one of the leading causes of cancer‐related death among males. An increasing number of men are diagnosed with this disease due to extensive serum prostate‐specific antigen (s‐PSA) testing. The heterogeneity of prostate carcinomas makes it important to improve prognostication and stratification to avoid over‐treatment of indolent cancers and identify the aggressive ones requiring radical treatment, systemic or targeted molecular therapy.

Forkhead box protein C2 (FOXC2) is an epithelial‐to‐mesenchymal transition (EMT)‐regulating transcription factor of the forkhead/winged helix‐family 1. EMT is a conserved embryonic cellular trans‐differentiation programme, seen in development and pathological conditions such as wound healing, fibrosis and in aggressive carcinomas 2, 3. In EMT, the polarised epithelial tumour cells shift to a motile, mesenchymal‐like phenotype, thereby enabling the tumour cells to become invasive and to metastasise 3, 4. During EMT, tumour cells experience loss of apical‐basal polarity due to reduced E‐cadherin (adherens junctions), gain of N‐cadherin, and loss of tight junctions and desmosomes. Tumour EMT is initiated and controlled by multiple signalling pathways, involving transforming growth factor β (TGFβ) 5, 6, and transcription factors Twist, Slug, Snai1, ZEB1 and others 5. However, there have been relatively few studies on EMT‐related markers and the progress and prognosis of prostate cancer 7, 8, 9, 10.

FOXC2 has been shown to induce EMT in cooperation with other EMT regulating transcription factors like Slug and Twist 1, and was newly brought to attention by the identification of a small molecule inhibiting this factor 11. FOXC2 seems to act as a mesenchymal inductor at a later stage during EMT, and its expression has been associated with aggressive human breast cancers 12. In non‐small‐cell lung carcinoma, FOXC2 mediates the transcriptional repression of p120‐catenin, which is a positive regulator of E‐cadherin 13. In gastric cancer, FOXC2 was found to positively regulate N‐cadherin expression by SENP3 14. Beyond its function as an EMT regulator, FOXC2 promotes cell proliferation and is a mediator of angiogenesis and lymphangiogenesis 15, 16.

Previous work in our group has demonstrated the prognostic value of the hallmark of EMT, an E‐cadherin to N‐cadherin switch (EN‐switch), in localised human prostate cancer 9. The cadherin switch has been described in other cancer types, such as malignant melanoma 17, breast cancer 18, extrahepatic cholangiocarcinoma 19 and ovarian high‐grade serous carcinoma 20, but the relevance of the intermediate stages within the spectrum of epithelial–mesenchymal plasticity has not been much studied in clinical materials of prostate cancer.

Up‐regulation of FOXC2 has been associated with progression and poor prognosis in various malignancies such as lung 21, 22, colon 23, gastric 24, oral tongue squamous cell carcinoma 25 and hepatocellular carcinomas 26. However, there are still no available survival data on FOXC2 in prostate cancer.

The aims of this study were to examine the expression of EMT‐related FOXC2, E‐cadherin and N‐cadherin in various prostatic tissues and to validate the results from our previous study on the E‐cadherin to N‐cadherin switch 9. We used a larger series with extended long‐term follow‐up and evaluated the clinico‐pathological correlates and relationship to patient survival. Here we provide a rationale for applying FOXC2 as a biomarker in prognostication and novel targeted therapy in prostate cancer and lend support to the significance of epithelial–mesenchymal phenotypes in localised and advanced disease.

## Materials and methods

### Patients and tissues

Series 1 is a retrospective, consecutive series of 338 patients (median age 61.0 years) treated by radical prostatectomy for clinically localised prostate cancer at Haukeland University Hospital, Bergen, Norway during 1986–2007, in part before the PSA screening era in Norway. This series has long and complete follow‐up, described previously for the first 104 cases 9, 27. A major part of this series is a clinically heterogeneous group of Gleason score 7 carcinomas (*n* = 199). Series 2–5 include 41 cases of benign prostatic hyperplasia (BPH), 33 non‐skeletal metastases (28 from lymph nodes, the others from rectum, orbita, subcutaneous tissue, testis and bronchial mucosa), 13 skeletal metastases from different patients, and prostate cancer tissues from 33 castration‐resistant prostate cancer (CRPC) patients (median age 77.3 years) harvested from the prostate by palliative transurethral resection during 1990–2005. The tumours in series 1 and 3–5 are predominantly acinar adenocarcinomas, including one mucinous adenocarcinoma among the CRPCs and an adenocarcinoma of foamy gland‐type among the non‐skeletal metastases. The non‐skeletal metastases also harboured an intermediate cell type variant of small cell carcinoma (one case). This study was approved by the Western Regional Committee for Medical and Health Research Ethics, REC West (REK 2015/2178).

### Clinico‐pathological variables

The following variables were recorded: age at diagnosis, preoperative s‐PSA, clinical stage (UICC TNM classification of malignant tumours, Eighth edition 28), Gleason grade and score, largest tumour diameter, extra‐prostatic extension (EPE), seminal vesicle invasion, involvement of surgical margins, and pelvic lymph node status at prostatectomy. Because of the important revisions in the recommendations from the International Society of Urological Pathology Consensus Conference in 2005 29, the slides from the radical prostatectomies were re‐examined and Gleason graded accordingly. In Norway, s‐PSA was introduced in the early 1990s. Hence, clinical stage T2 dominates in the first part of series 1, whereas clinical stage T1c dominates in the second part. Furthermore, the patients in the early group have larger tumours and more advanced pathological stages compared to the PSA‐detected tumours in the latter group.

### Follow‐up

For the 338 patients treated by radical prostatectomy, time from surgery until biochemical recurrence, clinical recurrence, loco‐regional recurrence, skeletal metastases and death (including cancer specific death) were recorded. Biochemical recurrence was defined as s‐PSA level of ≥0.5 ng/ml in two consecutive blood samples if the blood sample was taken before 31 December 1994. Due to the introduction of more sensitive s‐PSA measurements, all biochemical recurrences from 1 January 1995 were defined as s‐PSA level of ≥0.2 ng/ml in two consecutive blood samples. Loco‐regional recurrence was defined as a tumour in the prostatic fossa (confirmed by palpation/histology/cytology or radiology) or an elevation of s‐PSA following s‐PSA level <0.1 ng/ml or a >50% PSA‐drop after local radiation therapy. Skeletal metastases were found on bone scan, X‐ray or MRI. The last date of follow‐up was September 2016 and the median follow‐up time was 147.5 months. For surviving patients, median follow‐up time was 151.5 months. Two patients were lost to follow‐up, 169 patients experienced biochemical recurrence, 101 had clinical recurrence (41 with skeletal metastasis, 77 loco‐regional recurrences) and 38 patients died of prostate cancer. In nine patients diagnosed before the PSA era, disease progression was based on physical examination or imaging results.

Castration resistance (*n* = 33) was defined as disease progression during androgen‐ablation therapy. A total of 24% of the patients (8/33) received additional treatment with anti‐androgens (bicalutamide) prior to palliative transurethral resection. Most patients had clinical progression and increasing s‐PSA on consecutive measurements.

### Tissue microarrays

Three tissue cores (diameter 0.6–1.0 mm) were obtained from the area of highest tumour grade in each case and transferred to a recipient paraffin block. Tissue microarray (TMA) sections were used for all tissue subgroups apart from the 13 skeletal metastases.

### Immunohistochemistry

Staining was performed on sections (5 μm) from formalin‐fixed and paraffin‐embedded tissue.

#### Forkhead box protein C2

Sections were boiled in Tris‐EDTA buffer (pH 9.0) for 20 min at 350 W and incubated overnight at 4 °C with the monoclonal mouse antibody FOXC2 (M02), clone 2H3 (Abnova, Taipei, Taiwan) diluted 1:200 and stained with HRP EnVision mouse (DAKO, Glostrup, Denmark) for 30 min at room temperature.

#### E‐cadherin and N‐cadherin

Staining for E‐cadherin and N‐cadherin were performed according to previous protocols 9. For E‐cadherin, slides were boiled for 15 min at 350 W in citrate buffer (pH 6.0). The monoclonal mouse E‐cadherin antibody M3612 (diluted 1:400; DAKO) was used for incubation overnight at 4 °C. For N‐cadherin, after boiling in Tris‐EDTA buffer (pH 9.0) for 20 min at 350 W, sections were incubated with the monoclonal mouse antibody M3613 (diluted 1:25; DAKO) for 1 h at room temperature.

For all antibodies, the peroxidase was localised by the diaminobenzidine tetrachloride peroxidase reaction and counterstained with Mayer's haematoxylin. Negative controls were obtained using isotypic immunoglobulin and antibody diluent omitting the primary antibody. Samples, including multi‐organ TMA sections, with known reactivity were used as positive controls.

### Evaluation of staining in prostate tissues

The examination and scoring of the tissues were performed by one pathologist (AB), partly by two pathologists (AB, KG), blinded to patient characteristics and outcome.

**Figure 1:**
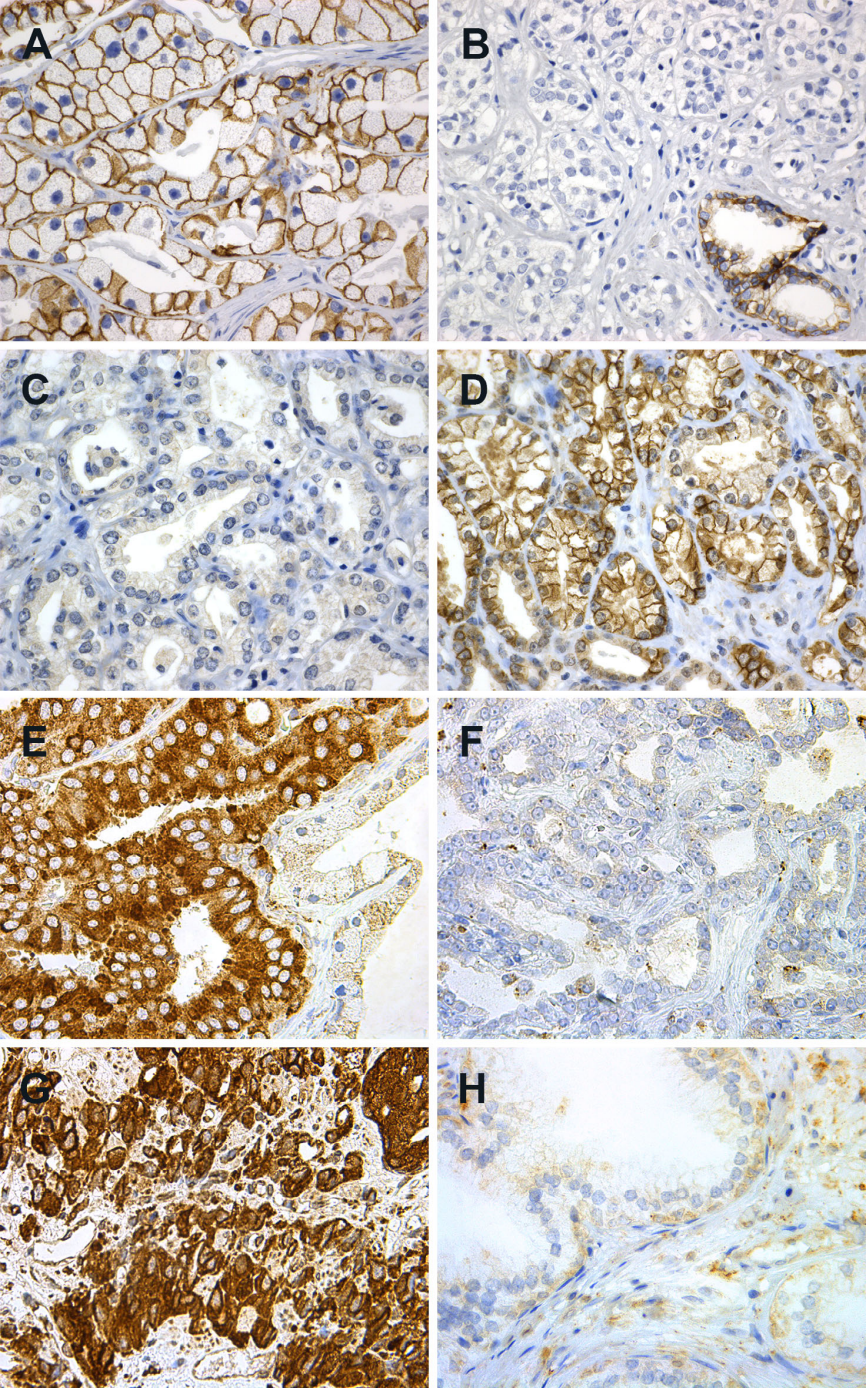
Immunohistochemical staining of (A–F) localised prostatic carcinomas, (G) castration resistant carcinoma and (H) BPH. (A) Strong membranous E‐cadherin; (B) weak E‐cadherin staining with strong staining in a benign gland at the lower right corner; (C) negative membranous N‐cadherin staining; (D) positive membranous N‐cadherin staining; (E) strong cytoplasmic FOXC2 in carcinoma, weaker staining in the benign epithelium to the right; (F) weak FOXC2 staining; (G) strong cytoplasmic FOXC2 staining in castration resistant carcinoma and (H) weak FOXC2 staining in BPH. Original magnification, ×400.

A semi‐quantitative and subjective grading system was used to record the staining; a staining index (SI; values 0–9) was calculated as a product of staining intensity (values 0–3) and proportion of positive tumour cells (0% = 0, 1–10% = 1, 11–50% = 2, >50% = 3) in all three tissue cores. E‐cadherin stained cell membranes in benign epithelium and variably cell membranes in malignant epithelium (Figure 1A,B). N‐cadherin stained both cytoplasm and cell membranes. The cytoplasmic staining was of variable intensity. Membranous N‐cadherin is presented in this study (Figure 1C,D). FOXC2 stained mostly the tumour cell cytoplasm (Figure 1E–H), while nuclear staining was weak and sporadic in all tissue groups. Cytoplasmic FOXC2 expression is presented in this study.

There are no standardised cut‐off values for any of the candidate biomarkers. Therefore, the frequency distribution of the markers, including median and quartile values, were examined, also considering the number of cases and events in each subgroup. Categories with similar survival estimates were merged when appropriate. To avoid cut‐off point selection bias, we aimed to use robust cut‐off values, such as the median. Hence, cytoplasmic FOXC2 and membranous E‐cadherin were dichotomised by median (SI ≥ 6 versus others). For membranous N‐cadherin, there was a high number (67%) of negative cases (i.e. SI = 0), consequently the cut‐off point was set as negative (SI = 0) or positive (SI ≥ 1) SI. The EN‐switch was defined as the subgroup with combined weak membranous E‐cadherin and positive membranous N‐cadherin expression. Co‐expression of E‐cadherin and N‐cadherin was defined as the subgroup with combined membranous E‐cadherin above median and positive membranous N‐cadherin expression.

### Observer variation for FOXC2, E‐cadherin and N‐cadherin

Inter‐observer variability was tested by two pathologists (AB and KG) by independent re‐evaluation, blinded to earlier registration and clinical data on the first part of series 1 (radical prostatectomies, *n* = 104), and series 2–5 (BPH, non‐skeletal metastases, skeletal metastases, and castration resistant carcinomas). The inter‐observer agreement for FOXC2 staining indices was good or very good for series 1 (*κ*‐value 0.91, dichotomised by median SI), and series 2–5 (*κ*‐values 0.70–0.87). The inter‐observer agreement for E‐cadherin was very good for series 1 (*κ*‐value 0.96, by median SI), and series 2–5 (*κ*‐values 0.85–0.87). Inter‐observer agreement for N‐cadherin was good, very good or perfect for series 1 (*κ*‐value 0.96, by negative/positive SI), and series 2–5 (*κ*‐values 0.63–1.0). Intra‐observer variability was tested in a blinded manner by one pathologist (AB) on a randomly selected subset of 85 cases (25%) from series 1 with very good intra‐observer agreement for FOXC2 (*κ*‐value 0.92, by median SI) and on 234 radical prostatectomies from the second part of series 1, with good intra‐observer agreement for E‐cadherin (*κ*‐value 0.74, by median SI) and N‐cadherin (*κ*‐value 0.89, by negative/positive SI).

### Biomarkers from previous studies

Biomarkers from previous studies 27, 30, 31, among them vascular proliferation index (the percentage of Ki67 positive vessels by dual staining for FVIII and Ki67), proliferating micro‐vessel density by FVIII/Ki67 co‐expression, VEGF‐A, proliferation of immature tumour vessels by Nestin/Ki67 co‐expression and Ki67 were included for comparison.

#### *FOXC2* gene expression

To validate our immunohistochemistry findings, an external gene expression cancer data set, GSE 25136, was downloaded from Gene Expression Omnibus, www.ncbi.nlm.nih.gov/geo 32. Clinical data on these patients were collected from Oncomine, www.oncomine.org 33. The data set included 79 men treated with radical prostatectomy between 1993 and 1999 at Memorial Sloan Kettering Cancer Center. The outcome was biochemical recurrence as classified by three consecutive increases in the serum level of PSA > 0.1 ng/ml after radical prostatectomy.

### Statistics

Associations between categorical and continuous variables were assessed by Pearson's chi‐square or Fisher's exact test, the Mann–Whitney *U* or Kruskal–Wallis tests, when appropriate. Univariate survival analyses were done by the product‐limit method (log‐rank test) and visualised by Kaplan–Meier plots. Multivariate survival analyses were done using the Cox' proportional hazards method and the likelihood ratio test, including variables with *p* < 0.15 from univariate analyses. Model assumptions were examined by log–log plots. Tests for possible interactions were performed. Inter‐ and intra‐observer agreements were evaluated by *κ*‐statistics. The SPSS statistical package versions 24.0 and 25.0 (IBM Corp., Armonk, NY, USA) were used.

## Results

### Biomarker expression in radical prostatectomies

In 199 Gleason score 7 carcinomas, 120 of 198 (61%) showed strong expression of FOXC2. E‐cadherin staining was weak in 42 of 198 (21%), N‐cadherin staining was positive in 69 of 198 (35%), and the EN‐switch, with combined weak E‐cadherin staining and positive N‐cadherin staining, was present in 20 of 198 (10%) of the cases. A similar distribution was observed in the complete series 1 (*n* = 338): 62, 23, 33 and 10%, respectively.

### Biomarker associations with clinico‐pathological variables

FOXC2 expression was not associated with any clinico‐pathological features in Gleason score 7 carcinomas or in series 1 (Table 1 and see supplementary material, Table S1).

**Table 1:** Associations between FOXC2, EN‐switch, E‐cadherin, N‐cadherin, clinico‐pathological features and selected biomarkers in 199 patients with clinically localised prostatic adenocarcinoma, Gleason score 7 (radical prostatectomies)

Variables	FOXC2*			EN‐switch†			E‐cadherin‡			N‐cadherin§		
	Low *n* (%)	High *n* (%)	*P* value¶	Absent *n* (%)	Present *n* (%)	*P* value¶	High *n* (%)	Low *n* (%)	*P* value¶	Low *n* (%)	High *n* (%)	*P* value¶
Gleason score**			0.277			0.148			0.131			0.664
3+4	54 (42)	74 (58)		118 (92)	10 (8)		105 (82)	23 (18)		82 (64)	46 (36)	
4+3	24 (34)	46 (66)		60 (86)	10 (14)		51 (73)	19 (27)		47 (67)	23 (33)	
Extra‐prostatic extension			0.678			0.011			0.181			0.741
Absent	42 (41)	61 (59)		98 (95)	5 (5)		85 (82)	18 (18)		66 (64)	37 (36)	
Present	36 (38)	59 (62)		80 (84)	15 (16)		71 (75)	24 (25)		63 (66)	32 (34)	
Seminal vesicle invasion			0.811			0.103			0.014			0.731
Absent	66 (40)	100 (60)		152 (92)	14 (8)		136 (82)	30 (18)		109 (66)	57 (34)	
Present	12 (38)	20 (62)		26 (81)	6 (19)		20 (62)	12 (38)		20 (63)	12 (37)	
Pathological stage††			0.867			0.011			0.042			0.530
pT2	40 (39)	63 (61)		98 (95)	5 (5)		87 (84)	16 (16)		65 (63)	38 (37)	
≥pT3	38 (40)	57 (60)		80 (84)	15 (16)		69 (73)	26 (27)		64 (67)	31 (33)	
Lymph node infiltration‡‡			0.520			0.010			0.044			0.120
Absent§§	78 (40)	118 (60)		178 (91)	18 (9)		156 (80)	40 (20)		129 (66)	67 (34)	
Present	0 (00)	2 (100)		0 (00)	2 (100)		0 (00)	2 (100)		0 (00)	2 (100)	
VEGF‐A			0.051¶¶			0.300			1.000			0.292
Low	18 (35)	34 (65)		41 (79)	11 (21)		35 (67)	17 (33)		34 (65)	18 (35)	
High	1 (7)	13 (93)		9 (64)	5 (36)		9 (64)	5 (36)		7 (50)	7 (50)	
Nestin‐Ki67***			0.248			0.026			0.065			0.233
Low	18 (45)	22 (55)		35 (85)	6 (15)		31 (76)	10 (24)		28 (68)	13 (32)	
High	8 (31)	18 (69)		16 (62)	10 (38)		14 (54)	12 (46)		14 (54)	12 (46)	

* Cytoplasmic expression, cut‐off by median.

† Subgroup with combined weak membranous E‐cadherin and positive membranous N‐cadherin expression.

‡ Membranous expression, cut‐off by median.

§ Membranous expression, cut‐off by negative/positive.

¶ Pearson's chi‐square or Fisher's exact test.

** Gleason score in radical prostatectomy specimens.

†† Pathological stage, UICC TNM Classification of malignant tumours, Eighth edition, 2017 28.

‡‡ Pelvic lymph node infiltration at radical prostatectomy.

§§ Includes cases without lymphadenectomy.

¶¶ Cut‐off by lower quartile.

*** Proliferating micro‐vessel density by dual Nestin/Ki67 staining.

Among Gleason score 7 carcinomas, the presence of an EN‐switch was associated with high pathological stage, EPE, large tumour diameter (Mann–Whitney, *p* = 0.021) and pelvic lymph node infiltration (Table 1). Weak E‐cadherin expression was associated with high pathological stage, seminal vesicle invasion and pelvic lymph node infiltration (Table 1).

Both the EN‐switch and weak E‐cadherin expression were associated with all adverse clinico‐pathological variables in series 1, as summarised in supplementary material, Table S1.

### Associations between biomarkers

Among Gleason score 7 cases, strong FOXC2 expression (above median SI) tended to be associated with the EN‐switch (*p* = 0.065). Moderate to strong FOXC2 above the lower quartile was significantly or borderline associated with high vascular proliferation index by FVIII/Ki67 co‐expression (Mann–Whitney, *p* = 0.031, *p* = 0.024), proliferating micro‐vessel density by FVIII/Ki67 (Mann–Whitney, *p* = 0.055, *p* = 0.031) and increased VEGF‐A, in Gleason score 7 cases and in series 1 (Table 1 and see supplementary material, Table S1). In both series, strong cytoplasmic FOXC2 expression was associated with the presence of nuclear expression of FOXC2 (*p* = 0.025 and *p* = 0.001, respectively).

In Gleason score 7 cases, the EN‐switch and weak E‐cadherin were associated with increased vascular proliferation by Nestin‐Ki67 (Table 1). In series 1, weak E‐cadherin expression and the EN‐switch were associated with maximum Nestin micro‐vessel density (Mann–Whitney, *p* = 0.042 and *p* = 0.003), increased vascular proliferation by Nestin‐Ki67 (see supplementary material, Table S1) and high tumour cell proliferation by Ki67 (Mann–Whitney *p* = 0.032 and *p* = 0.051). Additionally, the EN‐switch was associated with increased VEGF‐A in series 1 (see supplementary material, Table S1).

In both series, strong membranous N‐cadherin was associated with weak E‐cadherin. In series 1, positive N‐cadherin was related to increased vascular proliferation by Nestin‐Ki67 (*p* = 0.057) (see supplementary material, Table S1).

### Univariate survival analyses, Gleason score 7

**Figure 2:**
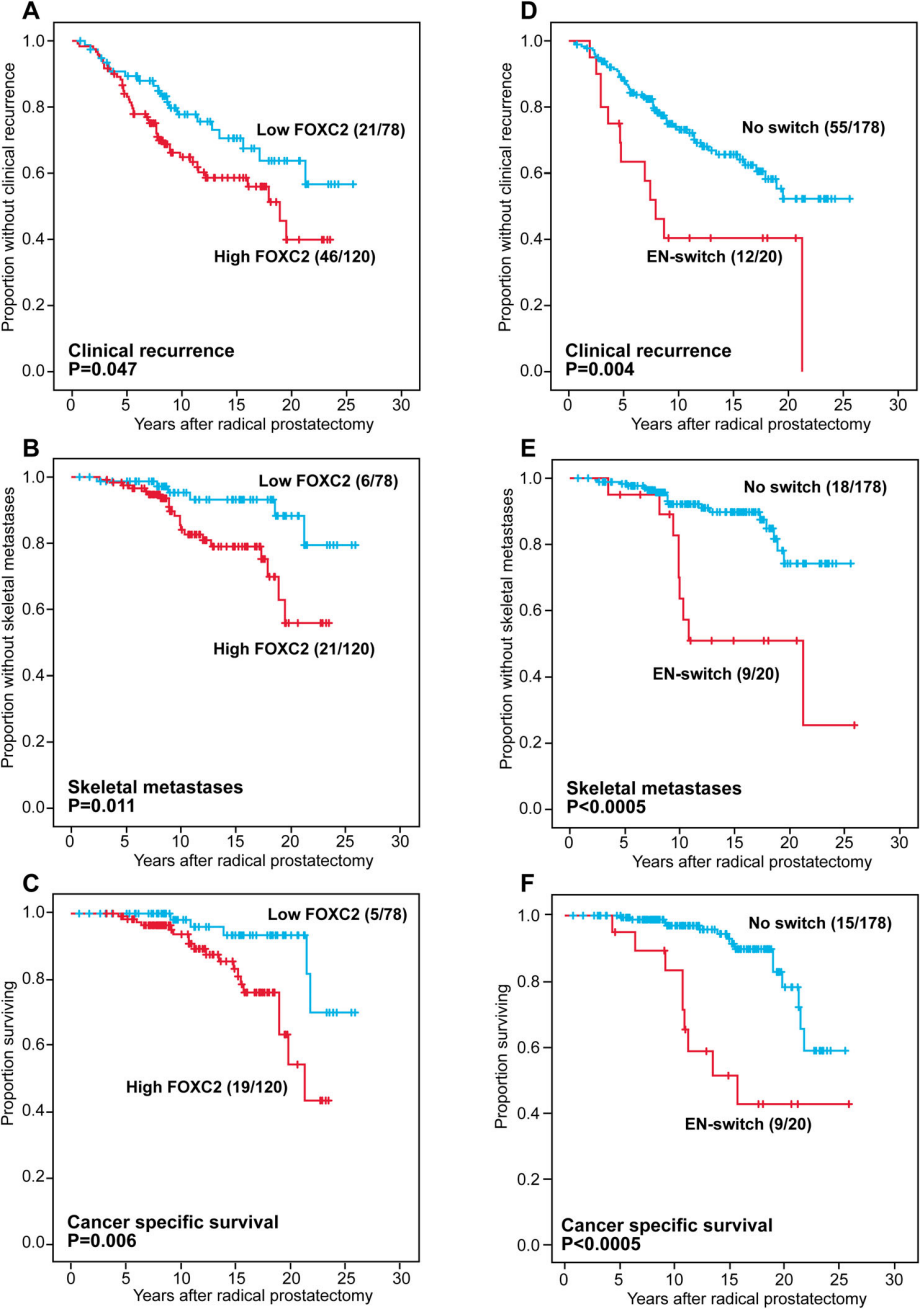
Univariate survival analyses (Kaplan–Meier) of Gleason score 7 prostate cancer patients after radical prostatectomy, by (A–C) cytoplasmic FOXC2 and (D–F) EN‐switch, with clinical recurrence, skeletal metastases and cancer specific death as end‐points.

In univariate survival analyses of Gleason score 7 patients, strong FOXC2 expression was significantly associated with clinical recurrence (*p* = 0.047), skeletal metastasis (*p* = 0.011) and cancer‐specific survival (*p* = 0.006) (Figure 2 and Table 2). Weak E‐cadherin expression and the presence of an EN‐switch were significantly associated with all end‐points, except for the EN‐switch versus biochemical recurrence (*p* = 0.061) (Figure 2 and Table 2).

**Table 2:** Univariate survival analysis (Kaplan–Meier) for 199 patients with Gleason score 7 carcinoma (radical prostatectomies), using biochemical recurrence, clinical recurrence, loco‐regional recurrence, skeletal metastasis and cancer specific death as end‐points

Variables	No. of patients	No. of events	Est. 5 years survival (%)	Est. 10 years survival (%)	Est. 15 years survival (%)	Est. 20 years survival (%)	*P* value*
Biochemical recurrence							
FOXC2†							0.174
Low	78	39	61.9	51.0	46.0	46.0	
High	120	71	54.7	38.2	36.1	31.6	
E‐cadherin‡							0.038
High	156	82	59.7	45.0	42.0	42.0	
Low	42	28	49.0	36.9	31.6	25.3	
N‐cadherin‡							0.739
Low	129	70	58.0	43.1	39.4	35.8	
High	69	40	56.3	43.1	40.2	40.2	
EN‐switch§							0.061
Absent	178	95	58.9	45.3	41.4	38.9	
Present	20	15	42.8	24.4	24.4	24.4	
Clinical recurrence							
FOXC2†							0.047
Low	78	21	89.4	77.8	70.6	63.7	
High	120	46	83.1	64.9	58.6	39.9	
E‐cadherin‡							0.016
High	156	47	89.4	74.2	65.7	51.8	
Low	42	20	70.5	53.2	53.2	47.9	
N‐cadherin‡							0.402
Low	129	40	87.2	72.0	64.7	51.3	
High	69	27	82.5	66.0	60.4	52.8	
EN‐switch§							0.004
Absent	178	55	87.9	73.1	65.6	52.3	
Present	20	12	63.5	40.4	40.4	40.4	
Loco‐regional recurrence							
FOXC2†							0.121
Low	78	16	90.7	82.4	77.6	70.2	
High	120	35	84.0	70.9	64.3	64.3	
E‐cadherin‡							0.018
High	156	35	90.1	80.0	72.4	68.1	
Low	42	16	73.0	58.7	58.7	58.7	
N‐cadherin‡							0.272
Low	129	29	88.8	78.0	70.2	70.2	
High	69	22	82.5	71.0	67.9	59.6	
EN‐switch§							<0.0005
Absent	178	40	89.0	79.5	72.8	69.0	
Present	20	11	63.5	40.4	40.4	40.4	
Skeletal metastasis							
FOXC2†							0.011
Low	78	6	98.7	95.3	93.2	88.3	
High	120	21	97.5	84.0	79.0	55.9	
E‐cadherin‡							0.004
High	156	15	99.3	93.2	90.4	72.5	
Low	42	12	92.7	73.0	65.9	65.9	
N‐cadherin‡							0.282
Low	129	14	97.6	90.9	89.1	72.2	
High	69	13	98.6	85.0	78.5	71.9	
EN‐switch§							<0.0005
Absent	178	18	98.3	92.2	89.8	74.2	
Present	20	9	95.0	63.6	50.9	50.9	
Cancer specific survival							
FOXC2†							0.006
Low	78	5	100.0	98.1	93.5	93.5	
High	120	19	99.2	93.7	83.3	54.4	
E‐cadherin‡							0.002
High	156	12	100.0	97.6	94.4	80.8	
Low	42	12	97.6	88.7	67.0	53.3	
N‐cadherin‡							0.756
Low	129	14	100.0	95.6	89.9	70.6	
High	69	10	98.6	95.1	83.8	80.2	
EN‐switch§							<0.0005
Absent	178	15	100.0	97.0	93.0	78.4	
Present	20	9	95.0	83.5	51.6	43.0	

* Log‐rank test.

† Cytoplasmic expression, cut‐off by median.

‡ Membranous expression, cut‐off by median (E‐cadherin) or negative/positive (N‐cadherin).

§ Subgroup with combined weak membranous E‐cadherin and positive membranous N‐cadherin expression.

### Multivariate survival analyses, Gleason score 7

In Cox' proportional hazards method of Gleason score 7 patients, the three standard prognostic variables Gleason score (4+3 versus 3+4), pathological stage (≥pT3 versus pT2) and preoperative s‐PSA (dichotomised by upper quartile; >13.3 versus ≤13.3) (see supplementary material, Table S2) were included in the model in addition to FOXC2 and the EN‐switch. FOXC2 was an independent predictor of time to clinical recurrence (HR 1.8, *p* = 0.023), skeletal metastasis (HR 3.7, *p* = 0.004) and cancer specific death (HR 4.8, *p* = 0.001), together with the EN‐switch and Gleason grade groups (GG3 [4+3] versus GG2 [3+4]) (Table 3).

**Table 3:** Multivariate survival analysis (Cox' proportional hazards method) for 199 patients with Gleason score 7 carcinoma (radical prostatectomies), using clinical recurrence, loco‐regional recurrence, skeletal metastasis and cancer specific death as end‐points

Variables	No. of patients	HR*	95% CI†	*P* value‡
Clinical recurrence				
Gleason score§				
3+4	127	1.0	1.4–3.8	
4+3	70	2.3		0.0010
Pathological stage¶				
pT2	102	1.0	1.0–2.9	
≥pT3	95	1.7		0.0340
FOXC2**				
Low	77	1.0	1.1–3.1	
High	120	1.8		0.0230
EN‐switch††				
Absent	177	1.0	1.0–3.8	
Present	20	2.0		0.0520
Loco‐regional recurrence				
Gleason score§				
3+4	128	1.0	1.2–3.8	
4+3	70	2.2		0.0060
Pathological stage¶				
pT2	103	1.0	1.2–4.1	
≥pT3	95	2.2		0.0090
EN‐switch††				
Absent	178	1.0	1.3–5.0	
Present	20	2.5		0.0150
Skeletal metastasis				
Gleason score§				
3+4	127	1.0	1.9–9.8	
4+3	70	4.3		<0.0005
FOXC2**				
Low	77	1.0	1.4–9.5	
High	120	3.7		0.0040
EN‐switch††				
Absent	177	1.0	1.7–8.7	
Present	20	3.8		0.0030
Cancer specific survival				
Gleason score§				
3+4	127	1.0	4.2–40.8	
4+3	70	13.2		<0.0005
Pathological stage¶				
pT2	102	1.0	1.8–21.2	
≥pT3	95	6.1		0.0010
FOXC2**				
Low	77	1.0	1.7–13.5	
High	120	4.8		0.0010
EN‐switch††				
Absent	177	1.0	2.7–18.7	
Present	20	7.0		<0.0005

* Hazard ratio.

† Confidence interval.

‡ Likelihood ratio test.

§ Gleason score in radical prostatectomy specimens.

¶ Pathological stage, UICC TNM Classification of malignant tumours, Eighth edition, 2017 28.

** Cytoplasmic staining, cut‐off by median.

†† Subgroup with combined weak membranous E‐cadherin and positive membranous N‐cadherin expression.

### Survival analyses across all Gleason scores

In univariate survival analyses including all 338 patients from series 1, strong FOXC2 expression was borderline associated with shorter time to clinical recurrence (*p* = 0.073), skeletal metastasis (*p* = 0.081), and cancer specific death (*p* = 0.069) (see supplementary material, Figure S1 and Table S3). The EN‐switch and weak E‐cadherin were strongly and significantly associated with all end‐points (see supplementary material, Figure S1 and Table S3). Positive N‐cadherin expression was associated with shorter time to skeletal metastases (*p* = 0.039).

Including all 338 patients from series 1, and variables from univariate survival analyses (see supplementary material, Tables S3 and S4) in multivariate Cox' regression analysis, the EN‐switch was an independent predictor of time to skeletal metastases (HR 3.7, *p* = 0.001) and cancer specific death (HR 8.4, *p* < 0.0005), together with FOXC2 (HR 1.9, *p* = 0.057 and HR 2.0, *p* = 0.061), Gleason grade groups (≥GG3 [4+3] versus ≤GG2 [3+4]) and pathological stage (≥pT3 versus pT2) (see supplementary material, Table S5).

### Survival analyses by *FOXC2* mRNA expression in radical prostatectomies

**Figure 3:**
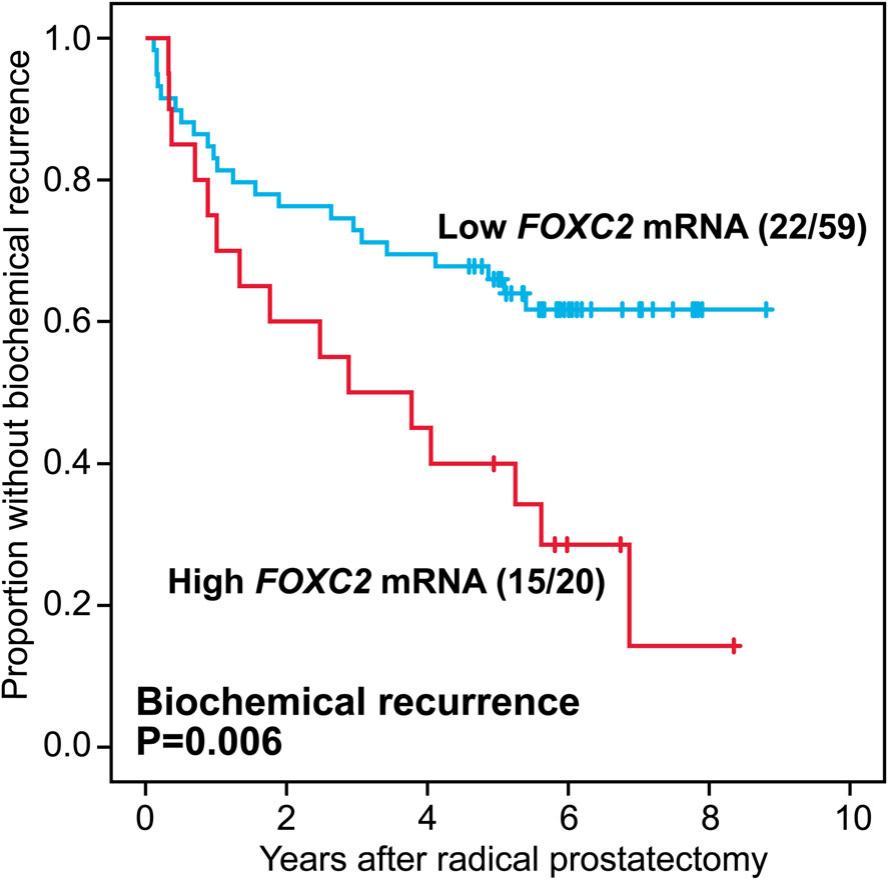
Univariate survival analysis (Kaplan–Meier) in 79 prostate cancer patients by *FOXC2* gene expression (59 low expressors, 20 high expressors) with biochemical recurrence as end‐point.

The 79 patients were divided into two groups with high (*n* = 20) and low (*n* = 59) *FOXC2* mRNA expression (upper quartile as cut‐point). Patients harbouring cancers with high *FOXC2* mRNA expression level showed shorter time to biochemical recurrence (*p* = 0.006) (Figure 3), and a similar result was observed among Gleason score 7 carcinomas (*n* = 44; *p* = 0.003).

### Biomarker expression in different prostatic tissues

#### FOXC2 expression

**Figure 4:**
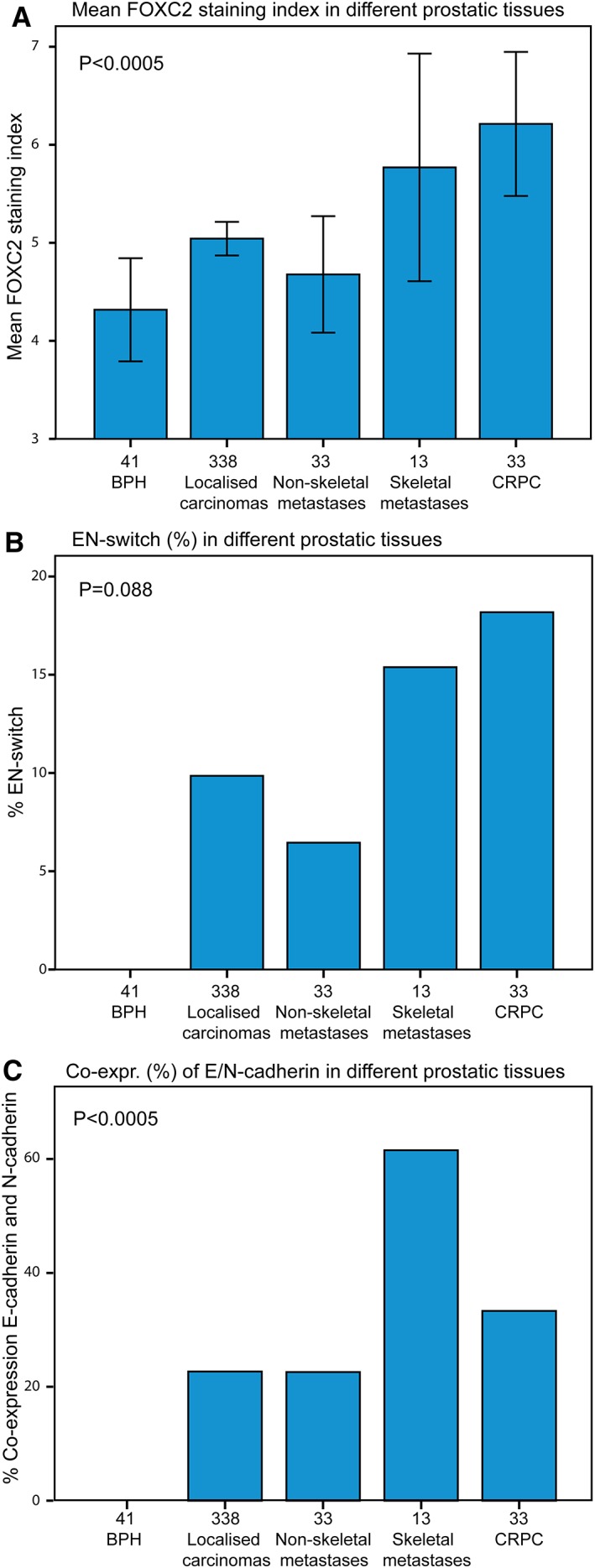
(A) Mean FOXC2 SI (95% CI) in benign and malignant prostatic tissues, (B) EN‐switch (%) in benign and malignant prostatic tissues, (C) co‐expression (%) of E‐cadherin and N‐cadherin in benign and malignant prostatic tissues.

FOXC2 staining was strongest among CRPC (median SI, 6; mean, 6.2), skeletal metastases (median SI, 6; mean, 5.8), localised carcinomas (median SI, 6; mean, 5.0) and non‐skeletal metastases (median SI, 6; mean, 4.7). In comparison, it was lowest among BPH (median SI, 3; mean, 4.3), (*p* < 0.0005) (Figure 4A).

#### E‐cadherin expression

E‐cadherin expression was lowest among CRPCs (median SI, 6; mean, 5.3) versus skeletal metastases (median SI, 6; mean, 6.2), localised cancers (median SI, 6; mean, 5.8) and non‐skeletal metastases (median SI, 6, mean, 5.7), and highest among hyperplasias (median SI, 6; mean, 7.4) (*p* < 0.0005).

#### N‐cadherin expression

N‐cadherin expression was most frequent and strongest among CRPCs (median SI, 1; mean, 1.8) and skeletal metastases (median SI, 2; mean, 1.3) in contrast to localised cancers (median SI, 0; mean, 0.7) and non‐skeletal metastases (median SI, 0; mean, 0.5), and not observed in BPH (*p* < 0.0005). In localised carcinomas, non‐skeletal metastases, skeletal metastases and CRPC, positive N‐cadherin expression was recorded in 33, 28, 77 and 52% of cases, respectively.

#### EN‐switch and cadherin co‐expression

The EN‐switch was most prevalent in castration resistant carcinomas (*n* = 6/33, 18.2%) (*p* = 0.006), skeletal metastases (*n* = 2/13, 15.4%) (*p* = 0.055) and localised carcinomas (*n* = 33/335, 9.9%) (*p* = 0.036), less frequently in non‐skeletal metastases (*n* = 2/31, 6.5%) (*p* = 0.182), as compared to hyperplasias (*n* = 0/41), in which the EN‐switch was not observed (Figure 4B). Co‐expression of E‐cadherin and N‐cadherin was most frequently observed in skeletal metastases (62%) and CRPC (33%) (*p* < 0.0005) (Figure 4C).

### Survival analyses in castration resistant carcinomas

**Figure 5:**
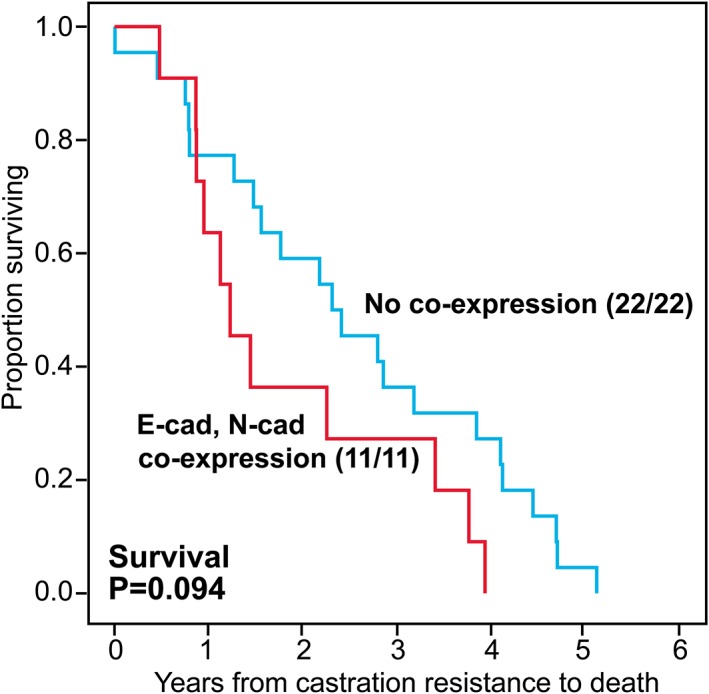
Univariate survival analysis (Kaplan–Meier) of castration resistant prostatic carcinomas by E‐cadherin and N‐cadherin co‐expression with time from diagnosis of castration resistance to death as end‐point.

Within the group of castration resistant cancers, no convincing survival differences were observed for FOXC2, E‐cadherin, N‐cadherin or the EN‐switch. However, co‐expression of E‐cadherin and N‐cadherin was borderline associated with reduced survival in these tumours (*p* = 0.094) (Figure 5), with time from castration resistance to death as the time variable.

## Discussion

Cadherin switching is a hallmark of EMT, important for tumour invasion and dissemination 3. FOXC2, an important EMT‐regulator 12, has recently been highlighted by the discovery of a novel small molecule inhibitor directly targeting this transcription factor 11. In our study, we have demonstrated that FOXC2 is strongly expressed in primary, metastatic and castration resistant prostate cancers, and is a strong predictor of outcome, of potential importance for prognostication and targeted therapy.

We previously reported that E‐ to N‐cadherin switching, an expression of EMT, was a strong and independent predictor of disease progression in prostate cancer 9, a finding that is currently sustained in the prolonged follow‐up and expansion of our series. We are aware of the limitations of retrospective studies and the heterogeneity among cases during the time period 1986–2007. However, the current series has a long follow‐up with solid end‐points. Still, the findings need to be validated in an independent cohort with comparable follow‐up. Validation in a more recent cohort of castration‐resistant prostate carcinomas treated with next generation anti‐androgen therapy, such as abiraterone and enzalutamide, would also be valuable. The sample sizes of series 1–5 are based on convenience and not powered to detect a pre‐specified difference.

FOXC2 has been introduced as a mediator of mesenchymal differentiation in the course of EMT, downstream of TGFβ and other EMT‐regulators, and identified as a decisive factor for carcinoma dissemination in breast cancer models 12. Increased expression of FOXC2 has been linked to oestrogen receptor negative basal‐like breast carcinoma 12 and survival in various cancer types 21, 22, 23, 24, 25, 26, but survival data for FOXC2 has not yet been presented in prostate cancer. A majority of clinically localised Gleason score 7 cancers are placed in the intermediate risk group (European Association of Urology 2017 34), a large and heterogeneous group of prostate cancers comprising Gleason grade groups 2 and 3. In order to better stratify intermediate risk patients to active surveillance or radical treatment, there is a need for biomarkers applicable to preoperative biopsy material. High expression of FOXC2 was a strong predictor of clinical recurrence and cancer specific survival among Gleason score 7 prostate cancer patients in our series, along with the EN‐switch and Gleason grade groups 3 versus 2. Thus, FOXC2 has the potential to serve as a biomarker of disease course, may impact surveillance and post‐operative follow‐up regimens, and ultimately direct tailored therapy.

Strong expression of FOXC2, the EN‐switch and co‐expression of E‐cadherin and N‐cadherin were less frequently observed in non‐skeletal metastases as compared to bone metastases. Prostate carcinomas favour bone marrow as their home for distant colonisation of tumour cells and form osteoblastic metastases. Tumour‐induced osteogenesis promotes growth of the metastatic tumour cells in the bone 35 and FOXC2 is found to induce osteogenesis 1. Hence, FOXC2 might be secreted by the carcinoma cells to induce an osteogenic metastatic niche in the bone marrow. This may even occur prior to the arrival of the disseminated tumour cells 36, 37. As lymphatic metastases may not require EMT 38, the differences in FOXC2 and cadherin expression between skeletal and non‐skeletal metastases might be explained by these aspects.

The EN‐switch was not a very prevalent finding among bone metastases and CRPC in our series, likely a reflection of the existence of intermediate states during EMT or mesenchymal‐to‐epithelial transition (MET) 2. Studies dealing with the concurrent immunohistochemical expression of E‐cadherin and N‐cadherin in tissue specimens from human prostate cancer metastases seem to be virtually non‐existent. The co‐expression of E‐cadherin and N‐cadherin in a majority of bone metastases thus corroborates previous experimental knowledge that partial reversion to an epithelial phenotype, i.e. partial MET, is necessary for colonisation to macrometastases at distant sites 39, possibly complicating reversal of EMT as a monotherapeutic approach 2. Co‐expression of E‐and N‐cadherin in a substantial proportion of the CRPCs in our series could be explained by partial EMT, with tumour cells transitioning between epithelial and mesenchymal states. It has earlier been reported that >80% of circulating tumour cells in patients with metastatic CRPC co‐express epithelial and mesenchymal markers 40. The phenotypic plasticity found in the advanced, castration resistant carcinomas and metastases in our material may be more important for the aggressiveness of carcinomas than the end states 40, 41. In support of this, a trend for reduced survival among our CRPC patients with a hybrid phenotype could be demonstrated. Furthermore, the frequent expression of N‐cadherin in metastatic and castration resistant disease may prove more important for therapeutic strategies than striving for a complete reversal of EMT. In line with this view, targeting N‐cadherin inhibits prostate cancer progression and castration resistance *in vivo* 42.

Cytoplasmic FOXC2 expression is presented in this study, comparable with immunohistochemical studies on FOXC2 in several other tumour types 12, 22, 24, 25, 26. Transcription factors may shuttle between the nucleus and the cytoplasm 43. The predominantly cytoplasmic expression of FOXC2 might indicate phenotypic plasticity with reversal of EMT in our material, since the balance between nuclear and cytoplasmic FOXC2 seems to determine whether FOXC2 acts to promote EMT or MET 43. Maintenance of FOXC2 in the cytoplasm of normal epithelial cells has been shown to be regulated through casein kinase 2‐mediated phosphorylation at serine 124 44. The newly described small molecule inhibitor of FOXC2 (MC‐1‐F2) induces degradation of FOXC2 and averts its nuclear localisation 11. Another therapeutic approach could be regulation of the shuttling of FOXC2 between the nucleus and the cytoplasm 45, for instance through targeting casein kinase 2 44.

Advanced prostate cancer patients receiving androgen deprivation therapy (ADT) eventually develop androgen resistance, which is a major determinant of prostate cancer mortality. ADT represents the standard treatment for advanced prostate cancer, but unfortunately induces EMT and cancer stem cell (CSC) like cells in prostate cancer 46, 47. The EMT programme is linked to the acquisition of CSC properties 48, which can be induced by FOXC2, leading to metastases as demonstrated in breast cancer cell line studies 49. The pivotal role of FOXC2 in prostate cancer was demonstrated by indirect suppression of FOXC2 in mice injected with a metastatic prostate cancer cell line, restoring epithelial phenotype, attenuating CSCs and, of major significance, resensitising prostate carcinoma cells to anti‐androgen and taxane therapy 50.

It has been appreciated that EMT induces resistance to chemotherapy and immunotherapy 2. Thus, it is exciting that a small molecule inhibitor of FOXC2 now has emerged. Administration of this inhibitor induced MET by reversal of E‐ to N‐cadherin switching in breast cancer cell lines expressing high levels of FOXC2, and reduced CSC properties and metastatic capabilities 11. On this background, our findings of highly increased FOXC2 expression in primary, metastatic and castration resistant prostatic carcinomas, with a strong link to poor outcome, make FOXC2 attractive for targeted therapy.

FOXC2 has also been described as a regulator of angiogenesis 15. In our study, FOXC2 was related to vascular factors, such as increased vascular proliferation index and VEGF‐A expression, supporting this theory. The EN‐switch was also related to vascular factors, suggesting that angiogenesis and EMT might be co‐regulated.

The major potential of FOXC2 as a therapy target lies not only in its role as an important downstream mediator of EMT, but is underscored by the recognition of its self‐inducing capacity through stimulation of known upstream regulators of EMT, among these TGFβ, Twist and others 12. However, EMT provides obstacles for effective hormonal intervention and chemotherapy in prostate cancer. Furthermore, a main challenge for blocking or reversing EMT in malignancies has been the inherent problems with targeting transcription factors, i.e. protein–protein interactions 51. Using the novel small molecule inhibitor of FOXC2 in prostate cancer overexpressors may possibly relieve these difficulties. Simultaneous targeting of EMT or its hybrid states, along with hormonal intervention and chemotherapy, may pave the way for multi‐modal treatment of prostate cancer in the future.

In conclusion, unravelling the expression levels of FOXC2 and the presence of epithelial–mesenchymal phenotypes in various settings of prostate cancer, and the relation to long term outcome, may have important implications for future prognostication, stratification and targeted therapy of prostate cancer patients.

## Author contributions statement

OJH and LAA designed the study. AB and KG performed the research. SAH and CB collected clinical data. All authors were involved in interpretation of data, data analysis, writing the paper and final approval of the submitted version.

## Supporting information


**Figure S1.** Univariate survival analyses (Kaplan–Meier) in 338 prostate cancer patients of all Gleason scores after radical prostatectomy

**Table S1.** Associations between FOXC2, EN‐switch, E‐cadherin, N‐cadherin, clinico‐pathological features and selected biomarkers in 338 patients with clinically localised prostatic adenocarcinoma (radical prostatectomies)
**Table S2.** Clinico‐pathological variables: univariate survival analysis (Kaplan–Meier) for 199 patients with Gleason score 7 carcinoma (radical prostatectomies)
**Table S3.** Univariate survival analysis (Kaplan–Meier) for 338 patients with adenocarcinoma (radical prostatectomies)
**Table S4.** Clinico‐pathological variables: univariate survival analysis (Kaplan–Meier) for 338 patients with adenocarcinoma (radical prostatectomies)
**Table S5.** Multivariate survival analysis (Cox' proportional hazards method) for 338 patients with adenocarcinoma (radical prostatectomies)
