# Supplementary material for: FOXC2 expression and epithelial–mesenchymal phenotypes are associated with castration resistance, metastasis and survival in prostate cancer
Source: J Pathol Clin Res. 2019 Oct 1;5(4):272–86. doi: 10.1002/cjp2.142 (PMC6817834; doi:10.1002/cjp2.142)
Supplement: Supplementary file 1 — Figure S1. Univariate survival analyses (Kaplan–Meier) in 338 prostate cancer patients of all Gleason scores after radical prostatectomy [file CJP2-5-272-s001.pdf]

FOXC2 expression and epithelial-mesenchymal phenotypes are associated with castration resistance, metastasis and survival in prostate cancer

Børretzen A *et al J Pathol Clin Res*, DOI 10.1002/cjp2.142

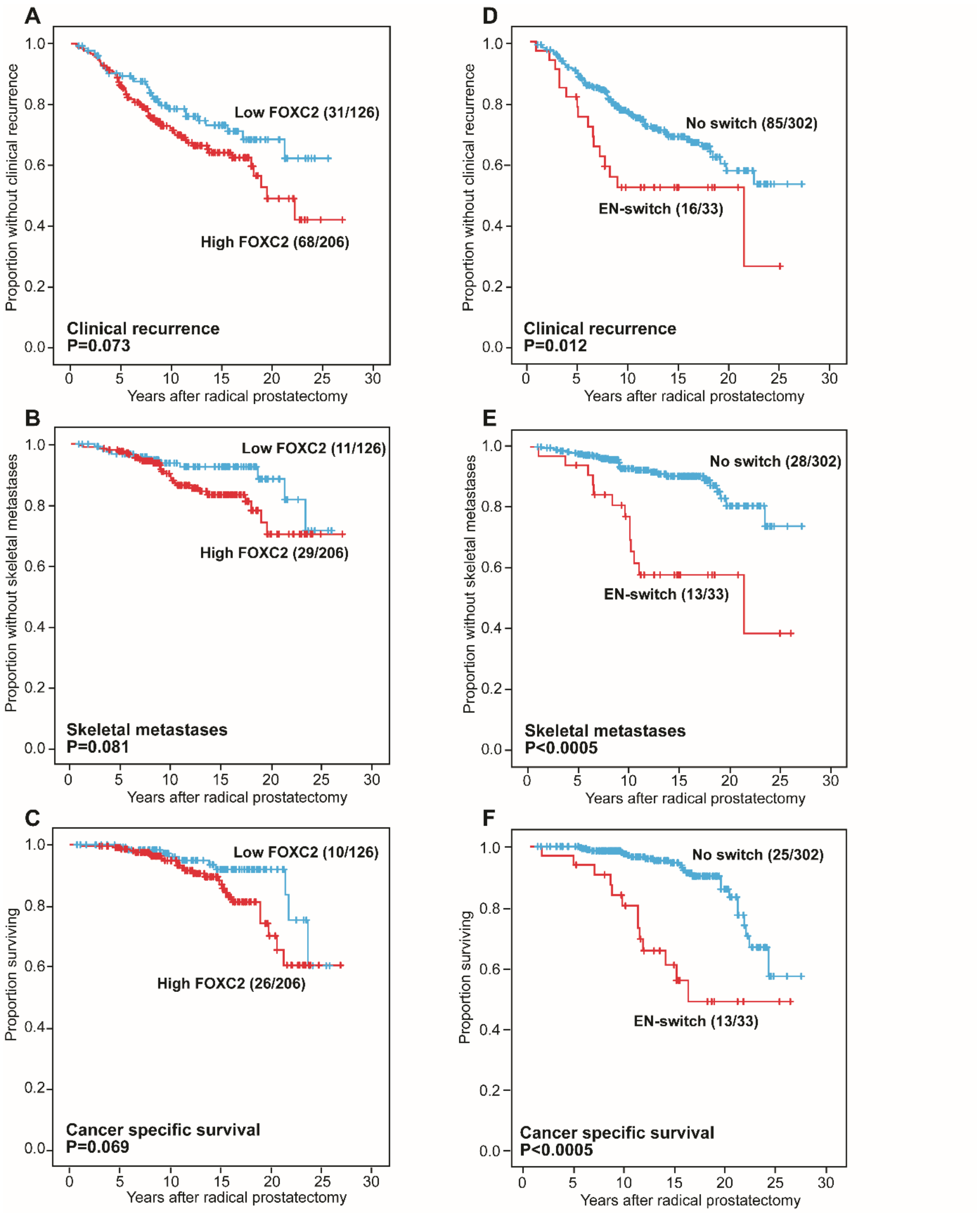

Figure S1. Univariate survival analyses (Kaplan-Meier) in 338 prostate cancer patients of all Gleason scores after radical prostatectomy, by (A-C) cytoplasmic FOXC2 and (D-F) EN-switch, with clinical recurrence, skeletal metastases and cancer specific death as end-points.
