# Supplementary material for: FOXC2 expression and epithelial–mesenchymal phenotypes are associated with castration resistance, metastasis and survival in prostate cancer
Source: J Pathol Clin Res. 2019 Oct 1;5(4):272–86. doi: 10.1002/cjp2.142 (PMC6817834; doi:10.1002/cjp2.142)
Supplement: Supplementary file 2 — Table S1. Associations between FOXC2, EN‐switch, E‐cadherin, N‐cadherin, clinico‐pathological features and selected biomarkers in 338 patients with clinically localised prostatic adenocarcinoma (radical prostatectomies) Table S2. Clinico‐pathological variables: univariate survival analysis (Kaplan–Meier) for 199 patients with Gleason score 7 carcinoma (radical prostatectomies) Table S3. Univariate survival analysis (Kaplan–Meier) for 338 patients with adenocarcinoma (radical prostatectomies) Table S4. Clinico‐pathological variables: univariate survival analysis (Kaplan–Meier) for 338 patients with adenocarcinoma (radical prostatectomies) Table S5. Multivariate survival analysis (Cox' proportional hazards method) for 338 patients with adenocarcinoma (radical prostatectomies) [file CJP2-5-272-s002.docx]

**FOXC2 expression and epithelial-mesenchymal phenotypes are associated with castration resistance, metastasis and survival in prostate cancer**

Børretzen A *et al J Pathol Clin Res,* DOI 10.1002/cjp2.142

| **Table S1.** Associations between FOXC2, EN-switch, E-cadherin, N-cadherin and clinico-pathological features and selected biomarkers in 338 patients with clinically localised prostatic adenocarcinoma (radical prostatectomies). | | | | | | | | | | | | | |
| --- | --- | --- | --- | --- | --- | --- | --- | --- | --- | --- | --- | --- | --- |
| **Variable** |  | **FOXC21** |  |  | **EN-switch2** | |  | **E-cadherin3** | |  | **N-cadherin4** | |  |
|  | Low n (%) | High n (%) | P-value5 | Absent n (%) | Present n (%) | | P-value5 | High n (%) | Low n (%) | P-value5 | Low n (%) | High n (%) | P-value5 |
| Gleason score6 |  |  | 0.276 |  |  | | 0.032 |  |  | <0.0005 |  |  | 0.963 |
| ≤3+4 | 91 (40) | 137 (60) |  | 211 (93) | 17 | (7) |  | 190 (83) | 38 (17) |  | 154 (68) | 74 (32) |  |
| ≥4+3 | 35 (34) | 69 (66) |  | 91 (85) | 16 (15) | |  | 69 (64) | 38 (36) |  | 72 (67) | 35 (33) |  |
| Extra-prostatic extension |  |  | 0.628 |  |  | | 0.004 |  |  | 0.013 |  |  | 0.798 |
| Absent | 78 (39) | 122 (61) |  | 188 (94) | 12 | (6) |  | 164 (82) | 36 (18) |  | 136 (68) | 64 (32) |  |
| Present | 48 (36) | 84 (64) |  | 114 (84) | 21 (16) | |  | 95 (70) | 40 (30) |  | 90 (67) | 45 (33) |  |
| Seminal Vesicle Invasion |  |  | 0.880 |  |  | | <0.0005 |  |  | 0.003 |  |  | 0.055 |
| Absent | 106 (38) | 172 (62) |  | 260 (93) | 20 | (7) |  | 225 (80) | 55 (20) |  | 195 (70) | 85 (30) |  |
| Present | 20 (37) | 34 (63) |  | 42 (76) | 13 (24) | |  | 34 (62) | 21 (38) |  | 31 (56) | 24 (44) |  |
| Pathological Stage7 |  |  | 0.888 |  |  | | 0.001 |  |  | 0.001 |  |  | 0.855 |
| pT2 | 75 (38) | 121 (62) |  | 186 (95) | 10 | (5) |  | 164 (84) | 32 (16) |  | 133 (68) | 63 (32) |  |
| ≥pT3 | 51 (38) | 85 (62) |  | 116 (84) | 23 (16) | |  | 95 (68) | 44 (32) |  | 93 (67) | 46 (33) |  |
| Lymph node infiltration8 |  |  | 0.260 |  |  | | 0.004 |  |  | 0.017 |  |  | 0.119 |
| Absent9 | 125 (39) | 200 (61) |  | 298 (91) | 29 | (9) |  | 256 (78) | 71 (22) |  | 223 (68) | 104 (32) |  |
| Present | 1 (14) | 6 (86) |  | 4 (50) | 4 (50) | |  | 3 (37) | 5 (63) |  | 3 (38) | 5 (62) |  |
| Ki67 |  |  | 0.887 |  |  | | 0.121 |  |  | 0.090 |  |  | 0.198 |
| Low | 16 (33) | 33 (67) |  | 43 (84) | 8 (16) | |  | 37 (72) | 14 (28) |  | 36 (71) | 15 (29) |  |
| High | 17 (34) | 33 (66) |  | 38 (72) | 15 (28) | |  | 30 (57) | 23 (43) |  | 31 (59) | 22 (41) |  |
| VEGF-A |  |  | 0.06210 |  |  | | 0.076 |  |  | 0.816 |  |  | 0.078 |
| Low | 23 (30) | 55 (70) |  | 67 (82) | 15 (18) | |  | 53 (65) | 29 (35) |  | 56 (68) | 26 (32) |  |
| High | 2 (10) | 19 (90) |  | 13 (62) | 8 (38) | |  | 13 (62) | 8 (38) |  | 10 (48) | 11 (52) |  |
| Nestin-Ki6711 |  |  | 0.304 |  |  | | <0.0005 |  |  | 0.001 |  |  | 0.057 |
| Low | 23 (37) | 39 (63) |  | 59 (89) | 7 (11) | |  | 50 (76) | 16 (24) |  | 47 (71) | 19 (29) |  |
| High | 10 (27) | 27 (73) |  | 22 (58) | 16 (42) | |  | 17 (45) | 21 (55) |  | 20 (53) | 18 (47) |  |
| 1Cytoplasmic expression, cut-off by median  2Subgroup with combined weak membranous E-cadherin and positive membranous N-cadherin expression  3Membranous expression, cut-off by median 4Membranous expression, cut-off by negative/positive 5Pearson’s Chi-square or Fisher’s Exact Test 6Gleason score in radical prostatectomy specimens  7Pathological stage, UICC TNM Classification of malignant tumours, Eighth edition, 2017  8Pelvic lymph node infiltration at radical prostatectomy  9Includes cases without lymphadenectomy  10Cut-off by lower quartile  11Proliferating microvessel density by dual Nestin/Ki67 staining | | | | | | |  |  |  |  |  |  |  |

**Table S2.** Clinico-pathological variables: Univariate survival analysis (Kaplan-Meier) for 199 patients with Gleason score 7 carcinoma (radical prostatectomies), using biochemical recurrence, clinical recurrence, loco-regional recurrence, skeletal metastasis and cancer specific death as end-points.

| **Variables** | **No. of**  **patients** | **No. of events** | **Est. 5 yrs. survival (%)** | **Est. 10 yrs. survival (%)** | **Est. 15 yrs. survival (%)** | **Est. 20 yrs.**  **survival (%)** | **P-value1** |
| --- | --- | --- | --- | --- | --- | --- | --- |
| **Biochemical recurrence** |  |  |  |  |  |  |  |
| Gleason score2 |  |  |  |  |  |  | <0.0005 |
| 3+4 | 129 | 59 | 71.4 | 52.7 | 47.4 | 44.4 |  |
| 4+3 | 70 | 51 | 32.9 | 26.9 | 26.9 | 26.9 |  |
| Pathological stage3 |  |  |  |  |  |  | <0.0005 |
| pT2 | 104 | 41 | 71.6 | 60.1 | 55.2 | 50.2 |  |
| ≥pT3 | 95 | 69 | 42.7 | 26.2 | 24.3 | 24.3 |  |
| Preoperative s-PSA4 |  |  |  |  |  |  | <0.0005 |
| Low | 139 | 63 | 66.4 | 53.5 | 49.6 | 46.0 |  |
| High | 56 | 43 | 32.6 | 23.9 | 21.5 | 21.5 |  |
| **Clinical recurrence** |  |  |  |  |  |  |  |
| Gleason score2 |  |  |  |  |  |  | <0.0005 |
| 3+4 | 129 | 32 | 92.7 | 79.6 | 73.1 | 53.5 |  |
| 4+3 | 70 | 35 | 72.9 | 53.0 | 46.1 | 42.8 |  |
| Pathological stage3 |  |  |  |  |  |  | 0.005 |
| pT2 | 104 | 23 | 88.0 | 80.7 | 74.0 | 60.1 |  |
| ≥pT3 | 95 | 44 | 82.8 | 59.1 | 52.6 | 42.5 |  |
| Preoperative s-PSA4 |  |  |  |  |  |  | 0.725 |
| Low | 139 | 42 | 85.3 | 71.0 | 64.5 | 55.8 |  |
| High | 56 | 22 | 85.1 | 66.4 | 61.3 | 52.5 |  |
| **Loco-regional recurrence** | | | | | | | |
| Gleason score2 |  |  |  |  |  |  | 0.002 |
| 3+4 | 129 | 24 | 92.7 | 81.5 | 76.6 | 73.7 |  |
| 4+3 | 70 | 27 | 75.7 | 64.8 | 57.0 | 52.9 |  |
| Pathological stage3 |  |  |  |  |  |  | 0.001 |
| pT2 | 104 | 15 | 89.0 | 86.5 | 82.1 | 82.1 |  |
| ≥pT3 | 95 | 36 | 83.9 | 64.2 | 57.2 | 52.4 |  |
| Preoperative s-PSA4 |  |  |  |  |  |  | 0.954 |
| Low | 139 | 34 | 86.0 | 77.0 | 70.2 | 63.8 |  |
| High | 56 | 15 | 87.0 | 71.8 | 69.2 | 69.2 |  |
| **Skeletal metastasis** |  |  |  |  |  |  |  |
| Gleason score2 |  |  |  |  |  |  | <0.0005 |
| 3+4 | 129 | 9 | 100.0 | 96.7 | 95.2 | 76.1 |  |
| 4+3 | 70 | 18 | 94.3 | 73.2 | 64.7 | 59.7 |  |
| Pathological stage3 |  |  |  |  |  |  | 0.095 |
| pT2 | 104 | 8 | 99.0 | 92.7 | 90.5 | 73.0 |  |
| ≥pT3 | 95 | 19 | 96.8 | 84.5 | 79.5 | 68.7 |  |
| Preoperative s-PSA4 |  |  |  |  |  |  | 0.343 |
| Low | 139 | 14 | 97.8 | 91.1 | 86.1 | 75.0 |  |
| High | 56 | 12 | 98.1 | 82.4 | 79.9 | 69.5 |  |
| **Cancer specific survival** |  |  |  |  |  |  |  |
| Gleason score2 |  |  |  |  |  |  | <0.0005 |
| 3+4 | 129 | 4 | 100.0 | 100.0 | 96.7 | 90.6 |  |
| 4+3 | 70 | 20 | 98.6 | 87.4 | 70.6 | 49.0 |  |
| Pathological stage3 |  |  |  |  |  |  | 0.003 |
| pT2 | 104 | 3 | 100.0 | 100.0 | 95.5 | 92.0 |  |
| ≥pT3 | 95 | 21 | 98.9 | 91.1 | 80.6 | 64.8 |  |
| Preoperative s-PSA4 |  |  |  |  |  |  | 0.149 |
| Low | 139 | 10 | 99.3 | 97.2 | 91.7 | 78.0 |  |
| High | 56 | 13 | 100.0 | 91.3 | 78.2 | 69.6 |  |
| 1Log-rank test  2Gleason score in radical prostatectomy specimens  3Pathological stage, UICC TNM Classification of malignant tumours, Eighth edition, 2017  4Preoperative s-PSA, dichotomised by upper quartile (>13.3 vs. ≤13.3) | | | | |  |  |  |

**Table S3.** Univariate survival analysis (Kaplan-Meier) for 338 patients with adenocarcinoma (radical prostatectomies), using biochemical recurrence, clinical recurrence, loco-regional recurrence, skeletal metastasis and cancer specific death as end-points.

| **Variables** | **No. of patients** | **No. of events** | **Est. 5 yrs. survival (%)** | **Est. 10 yrs. survival (%)** | **Est. 15 yrs. survival (%)** | **Est. 20 yrs. survival (%)** | **P-value1** |
| --- | --- | --- | --- | --- | --- | --- | --- |
| **Biochemical recurrence** | | | | | | | |
| FOXC22 |  |  |  |  |  |  | 0.419 |
| Low | 126 | 59 | 65.8 | 53.2 | 49.1 | 49.1 |  |
| High | 206 | 107 | 62.0 | 48.9 | 45.0 | 38.7 |  |
| E-cadherin3 |  |  |  |  |  |  | 0.017 |
| High | 259 | 122 | 65.5 | 53.0 | 48.6 | 45.3 |  |
| Low | 76 | 47 | 53.5 | 39.7 | 37.3 | 33.6 |  |
| N-cadherin3 |  |  |  |  |  |  | 0.526 |
| Low | 226 | 111 | 64.8 | 51.3 | 46.2 | 40.8 |  |
| High | 109 | 58 | 58.6 | 47.1 | 45.3 | 45.3 |  |
| EN-switch4 |  |  |  |  |  |  | 0.006 |
| Absent | 302 | 145 | 64.7 | 52.6 | 48.1 | 44.1 |  |
| Present | 33 | 24 | 44.6 | 27.0 | 27.0 | 27.0 |  |
| **Clinical recurrence** |  |  |  |  |  |  |  |
| FOXC22 |  |  |  |  |  |  | 0.073 |
| Low | 126 | 31 | 89.3 | 78.4 | 73.1 | 68.4 |  |
| High | 206 | 68 | 85.8 | 72.1 | 64.0 | 48.9 |  |
| E-cadherin3 |  |  |  |  |  |  | 0.033 |
| High | 259 | 70 | 88.5 | 78.0 | 69.8 | 56.7 |  |
| Low | 76 | 31 | 81.3 | 60.6 | 58.1 | 54.9 |  |
| N-cadherin3 |  |  |  |  |  |  | 0.341 |
| Low | 226 | 63 | 87.6 | 75.3 | 68.1 | 58.6 |  |
| High | 109 | 38 | 85.3 | 71.3 | 65.0 | 54.2 |  |
| EN-switch4 |  |  |  |  |  |  | 0.012 |
| Absent | 302 | 85 | 88.1 | 76.4 | 68.7 | 57.5 |  |
| Present | 33 | 16 | 75.3 | 52.0 | 52.0 | 52.0 |  |
| **Loco-regional recurrence** | | | | | | | |
| FOXC22 |  |  |  |  |  |  | 0.102 |
| Low | 126 | 23 | 91.8 | 83.8 | 79.7 | 74.7 |  |
| High | 206 | 52 | 86.7 | 77.6 | 70.3 | 66.8 |  |
| E-cadherin3 |  |  |  |  |  |  | 0.049 |
| High | 259 | 53 | 90.0 | 83.0 | 76.1 | 70.6 |  |
| Low | 76 | 24 | 82.6 | 67.6 | 65.0 | 65.0 |  |
| N-cadherin3 |  |  |  |  |  |  | 0.616 |
| Low | 226 | 49 | 89.0 | 79.6 | 73.2 | 73.2 |  |
| High | 109 | 28 | 87.1 | 78.9 | 74.1 | 61.9 |  |
| EN-switch4 |  |  |  |  |  |  | 0.040 |
| Absent | 302 | 65 | 89.8 | 81.4 | 74.8 | 70.4 |  |
| Present | 33 | 12 | 75.3 | 61.5 | 61.5 | 61.5 |  |
| **Skeletal metastasis** |  |  |  |  |  |  |  |
| FOXC22 |  |  |  |  |  |  | 0.081 |
| Low | 126 | 11 | 96.7 | 93.7 | 92.5 | 88.5 |  |
| High | 206 | 29 | 97.6 | 88.0 | 83.3 | 70.3 |  |
| E-cadherin3 |  |  |  |  |  |  | 0.003 |
| High | 259 | 23 | 98.4 | 93.8 | 90.8 | 78.8 |  |
| Low | 76 | 18 | 93.3 | 77.6 | 74.0 | 74.0 |  |
| N-cadherin3 |  |  |  |  |  |  | 0.039 |
| Low | 226 | 21 | 97.7 | 93.0 | 90.4 | 80.1 |  |
| High | 109 | 20 | 96.3 | 84.0 | 79.9 | 74.9 |  |
| EN-switch4 |  |  |  |  |  |  | <0.0005 |
| Absent | 302 | 28 | 97.6 | 92.9 | 90.3 | 80.5 |  |
| Present | 33 | 13 | 93.9 | 65.5 | 57.8 | 57.8 |  |
| **Cancer specific survival** | | | | | | | |
| FOXC22 |  |  |  |  |  |  | 0.069 |
| Low | 126 | 10 | 99.2 | 96.0 | 91.9 | 91.9 |  |
| High | 206 | 26 | 99.0 | 94.8 | 88.2 | 69.9 |  |
| E-cadherin3 |  |  |  |  |  |  | 0.002 |
| High | 259 | 20 | 99.6 | 97.5 | 95.1 | 84.6 |  |
| Low | 76 | 18 | 97.4 | 86.7 | 73.3 | 64.6 |  |
| N-cadherin3 |  |  |  |  |  |  | 0.180 |
| Low | 226 | 21 | 100.0 | 96.5 | 92.8 | 79.0 |  |
| High | 109 | 17 | 97.2 | 91.7 | 82.9 | 80.3 |  |
| EN-switch4 |  |  |  |  |  |  | <0.0005 |
| Absent | 302 | 25 | 99.7 | 96.6 | 93.8 | 83.3 |  |
| Present | 33 | 13 | 93.9 | 80.5 | 55.9 | 48.9 |  |
| 1Log-rank test  2Cytoplasmic expression, cut-off by median  3Membranous expression, cut-off by median (E-cadherin) or negative/positive (N-cadherin)  4Subgroup with combined weak membranous E-cadherin and positive membranous N-cadherin expression | | | | | | | |

**Table S4.** Clinico-pathological variables: Univariate survival analysis (Kaplan-Meier) for 338 patients with adenocarcinoma (radical prostatectomies), using biochemical recurrence, clinical recurrence, loco-regional recurrence, skeletal metastasis and cancer-specific death as end-points.

| **Variables** | **No. of patients** | **No. of events** | **Est. 5 yrs. survival (%)** | **Est. 10 yrs. survival (%)** | **Est. 15 yrs. survival (%)** | **Est. 20 yrs. survival (%)** | **P-value1** |
| --- | --- | --- | --- | --- | --- | --- | --- |
| **Biochemical recurrence** |  |  |  |  |  |  |  |
| Gleason score2 |  |  |  |  |  |  | <0.0005 |
| ≤3+4 | 231 | 87 | 77.9 | 63.6 | 57.9 | 52.7 |  |
| ≥4+3 | 107 | 82 | 31.8 | 22.6 | 22.6 | 22.6 |  |
| Pathological stage3 |  |  |  |  |  |  | <0.0005 |
| pT2 | 199 | 67 | 77.0 | 67.1 | 61.3 | 59.0 |  |
| ≥pT3 | 139 | 102 | 43.6 | 27.0 | 25.7 | 21.8 |  |
| Preoperative s-PSA4 |  |  |  |  |  |  | <0.0005 |
| Low | 250 | 103 | 70.8 | 59.2 | 54.3 | 51.0 |  |
| High | 82 | 60 | 38.0 | 26.7 | 24.9 | 24.9 |  |
| **Clinical recurrence** |  |  |  |  |  |  |  |
| Gleason score2 |  |  |  |  |  |  | <0.0005 |
| ≤3+4 | 231 | 44 | 93.2 | 85.7 | 79.6 | 65.3 |  |
| ≥4+3 | 107 | 57 | 73.8 | 50.0 | 41.9 | 37.3 |  |
| Pathological stage3 |  |  |  |  |  |  | <0.0005 |
| pT2 | 199 | 35 | 91.2 | 85.5 | 80.9 | 68.3 |  |
| ≥pT3 | 139 | 66 | 81.0 | 58.5 | 49.4 | 41.8 |  |
| Preoperative s-PSA4 |  |  |  |  |  |  | 0.093 |
| Low | 250 | 64 | 88.1 | 77.2 | 71.2 | 62.6 |  |
| High | 82 | 33 | 82.4 | 65.5 | 58.5 | 52.0 |  |
| **Loco-regional recurrence** | | | | | | | |
| Gleason score2 |  |  |  |  |  |  | <0.0005 |
| ≤3+4 | 231 | 35 | 93.2 | 87.3 | 82.0 | 80.0 |  |
| ≥4+3 | 107 | 42 | 78.4 | 62.8 | 55.4 | 49.4 |  |
| Pathological stage3 |  |  |  |  |  |  | <0.0005 |
| pT2 | 199 | 27 | 91.7 | 88.5 | 84.9 | 80.2 |  |
| ≥pT3 | 139 | 50 | 83.9 | 67.0 | 58.3 | 54.5 |  |
| Preoperative s-PSA4 |  |  |  |  |  |  | 0.326 |
| Low | 250 | 52 | 89.3 | 82.0 | 75.8 | 68.3 |  |
| High | 82 | 23 | 85.0 | 71.7 | 68.0 | 68.0 |  |
| **Skeletal metastasis** |  |  |  |  |  |  |  |
| Gleason score2 |  |  |  |  |  |  | <0.0005 |
| ≤3+4 | 231 | 10 | 100.0 | 97.8 | 97.0 | 84.3 |  |
| ≥4+3 | 107 | 31 | 91.6 | 72.7 | 64.3 | 61.1 |  |
| Pathological stage3 |  |  |  |  |  |  | <0.0005 |
| pT2 | 199 | 8 | 99.5 | 96.6 | 95.6 | 86.2 |  |
| ≥pT3 | 139 | 33 | 94.2 | 80.9 | 75.2 | 68.0 |  |
| Preoperative s-PSA4 |  |  |  |  |  |  | 0.003 |
| Low | 250 | 19 | 98.0 | 93.1 | 90.6 | 84.5 |  |
| High | 82 | 20 | 94.9 | 81.5 | 76.4 | 69.2 |  |
| **Cancer specific survival** |  |  |  |  |  |  |  |
| Gleason score2 |  |  |  |  |  |  | <0.0005 |
| ≤3+4 | 231 | 5 | 100.0 | 100.0 | 97.2 | 93.4 |  |
| ≥4+3 | 107 | 33 | 97.2 | 83.7 | 72.3 | 55.4 |  |
| Pathological stage3 |  |  |  |  |  |  | <0.0005 |
| pT2 | 199 | 3 | 100.0 | 100.0 | 97.9 | 96.2 |  |
| ≥pT3 | 139 | 35 | 97.8 | 88.0 | 78.7 | 65.5 |  |
| Preoperative s-PSA4 |  |  |  |  |  |  | 0.003 |
| Low | 250 | 15 | 99.6 | 96.8 | 94.2 | 87.2 |  |
| High | 82 | 21 | 97.5 | 88.7 | 78.5 | 68.4 |  |
| 1Log-rank test  2Gleason score in radical prostatectomy specimens  3Pathological stage, UICC TNM Classification of malignant tumours, Eighth edition, 2017  4Preoperative s-PSA, dichotomised by upper quartile (>13.3 vs. ≤13.3) | | | | |  |  |  |

**Table S5.** Multivariate survival analysis (Cox’ proportional hazards method) for 338 patients with adenocarcinoma (radical prostatectomies), using clinical recurrence, loco-regional recurrence, skeletal metastasis and cancer specific death as end-points.

| **Variables** | **No. of patients** | **HR1** | **95 % CI2** | **P-value3** |
| --- | --- | --- | --- | --- |
| **Clinical recurrence** |  |  |  |  |
| Gleason score4 |  |  |  |  |
| ≤3+4 | 228 | 1.0 |  |  |
| ≥4+3 | 104 | 2.8 | 1.8-4.2 | <0.0005 |
| Pathological stage5 |  |  |  |  |
| pT2 | 196 | 1.0 |  |  |
| ≥pT3 | 136 | 2.2 | 1.4-3.3 | <0.0005 |
| FOXC26 |  |  |  |  |
| Low | 126 | 1.0 |  |  |
| High | 206 | 1.6 | 1.0-2.4 | 0.037 |
| **Loco-regional recurrence** |  |  |  |  |
| Gleason score4 |  |  |  |  |
| ≤3+4 | 228 | 1.0 |  |  |
| ≥4+3 | 104 | 2.4 | 1.5-3.9 | <0.0005 |
| Pathological stage5 |  |  |  |  |
| pT2 | 196 | 1.0 |  |  |
| ≥pT3 | 136 | 2.2 | 1.3-3.6 | 0.002 |
| FOXC26 |  |  |  |  |
| Low | 126 | 1.0 |  |  |
| High | 206 | 1.6 | 1.0-2.6 | 0.069 |
| **Skeletal metastasis** |  |  |  |  |
| Gleason score4 |  |  |  |  |
| ≤3+4 | 226 | 1.0 |  |  |
| ≥4+3 | 104 | 6.2 | 3.0-12.8 | <0.0005 |
| Pathological stage5 |  |  |  |  |
| pT2 | 194 | 1.0 |  |  |
| ≥pT3 | 136 | 3.1 | 1.4-6.9 | 0.002 |
| EN-switch7 |  |  |  |  |
| Absent | 297 | 1.0 |  |  |
| Present | 33 | 3.7 | 1.8-7.3 | 0.001 |
| FOXC26 |  |  |  |  |
| Low | 124 | 1.0 |  |  |
| High | 206 | 1.9 | 1.0-4.0 | 0.057 |
| **Cancer specific survival** |  |  |  |  |
| Gleason score4 |  |  |  |  |
| ≤3+4 | 226 | 1.0 |  |  |
| ≥4+3 | 104 | 16.9 | 6.0-47.3 | <0.0005 |
| Pathological stage5 |  |  |  |  |
| pT2 | 194 | 1.0 |  |  |
| ≥pT3 | 136 | 9.1 | 2.7-30.3 | <0.0005 |
| EN-switch7 |  |  |  |  |
| Absent | 297 | 1.0 |  |  |
| Present | 33 | 8.4 | 3.7-19.3 | <0.0005 |
| FOXC26 |  |  |  |  |
| Low | 124 | 1.0 |  |  |
| High | 206 | 2.0 | 0.9-4.2 | 0.061 |
| ^1^Hazard ratio  ^2^Confidence interval  ^3^Likelihood ratio test  ^4^Gleason score in radical prostatectomy specimens  ^5^Pathologic stage, UICC TNM Classification of malignant tumours, Eighth edition, 2017  ^6^Cytoplasmic staining, cut-off by median  ^7^Subgroup with combined weak membranous E-cadherin and positive membranous N-cadherin expression | | | | |
